# Supplementary material for: Education Practices of Dietitians Across Australia and New Zealand Around the Glycaemic Management of Dietary Fat and Protein in Type 1 Diabetes and the Use of Continuous Glucose Monitoring: A Survey Evaluation
Source: Nutrients. 2025 Mar 22;17(7):1109. doi: 10.3390/nu17071109 (PMC11990433; doi:10.3390/nu17071109)
Supplement: Supplementary file 1 [file nutrients-17-01109-s001.zip › nutrients-3487150-supplementary.pdf]

A survey-based evaluation of the current education practices of dietitians across Australia and New Zealand

Please read the statement below:

I agree to participate in the anonymous online survey. I understand that the project will be conducted as described in the Information Statement I have been provided, a copy of which I have retained. I understand that I can withdraw at any time without giving a reason before submitting my survey, I understand that after I submit my survey this will not be possible. I understand that the information I provide cannot be linked to myself, or my clinic. I have had the opportunity to have any questions answered to my satisfaction.

Please select one of the following consent options:

◯ I agree to participate in the survey
◯ I do not agree to participate in the survey

Welcome! Let's get started. The following section focuses on your clinic characteristics, carbohydrate counting education and insulin dosing. It is estimated that this section will take less than 5 minutes to complete.

How many years have you been working in type 1 diabetes management?

________________________________ Discrete

Who are the members of your diabetes team?

Please check all that apply.

- ☐ Dietitian
- ☐ Diabetes Educator
- ☐ Endocrinologist
- ☐ Paediatrician
- ☐ Psychologist
- ☐ Social Worker
- ☐ Other

Categorical

Please specify the full-time equivalent (FTE) for the dietitian.

________________________________ Continuous

Please specify how many children aged 0-17.9 years with type 1 diabetes attend your clinic.

________________________________ Discrete

In what state and/ country is your clinic located?

- ◯ New Zealand
- ◯ ACT-Australia
- ◯ NT-Autralia
- ◯ NSW-Australia
- ◯ QLD-Australia
- ◯ SA-Australia
- ◯ TAS-Australia
- ◯ VIC-Australia
- ◯ WA-Australia

Categorical

In what area is your clinic located?

- ◯ metropolitan
- ◯ regional
- ◯ rural

Categorical

What proportion of children attending your clinic are currently using CGM?

- ◯ None
- ◯ ≤25%
- ◯ >25%-50%
- ◯ >50%-75%
- ◯ >75%-100%

Categorical

Do you teach children using insulin pump therapy to count carbohydrate?

- ◯ Yes, I teach all children (100%) using insulin pump therapy
- ◯ Yes, I teach most children (>50%) using insulin pump therapy
- ◯ Yes, I teach select children (≤50%) using insulin pump therapy
- ◯ No, I do not teach children using insulin pump therapy

Categorical

How do you teach children using insulin pump therapy to count carbohydrate?

- ◯ 1g
- ◯ 10g portions
- ◯ 15g exchanges
- ◯ Cup/ hand measure

Categorical

Do you teach children using insulin injections to count carbohydrate?

- ◯ Yes, I teach all children (100%)
- ◯ Yes, I teach most children (>50%)
- ◯ Yes, I teach select children (≤50%)
- ◯ No, I do not teach children

Categorical

How do you teach children using insulin injections to count carbohydrate?	◯ 1g<br>◯ 10g portions<br>◯ 15g exchanges<br>◯ Cup/ hand measure	Categorical
Do you start children using insulin injections on an insulin to carb ratio (ICR)?	◯ Yes<br>◯ No	Categorical
When do you start children using insulin injections on an insulin to carb ratio (ICR)?	◯ At diagnosis<br>◯ At the first clinic visit<br>◯ Within the first 6 months of diagnosis<br>◯ Within the first year of diagnosis<br>◯ When the team decides	Categorical

Categorical

Great work! You have completed section 1/3 of the survey. The following section focuses on fat and protein education and insulin dosing. It is estimated that this section will take less than 10 minutes to complete.

Question	Response
Do you think that providing education on the glycaemic impact of fat and protein and management strategies is part of your role as a paediatric diabetes dietitian?	◯ Yes<br>◯ No<br>◯ Not sure
Do you teach families to identify fat and protein foods?	◯ Yes, I teach all families (100%)<br>◯ Yes, I teach most families (>50%)<br>◯ Yes, I teach select families (≤50%)<br>◯ No, I do not teach families
How do you teach families to identify fat and protein foods?	◯ 1g increments<br>◯ Thresholds i.e. count if >20g<br>◯ Ranges i.e., high, medium and low<br>◯ Food groups i.e., dairy, meat<br>◯ Other
Please describe the other method you use to teach families to identify fat and protein foods.	__________________________________
Do you educate families on the glycaemic impact of fat and protein?	◯ Yes, I provide education to all (100%) families<br>◯ Yes, I provide education to most (>50%) families<br>◯ Yes, I provide education to select (≤50%) families<br>◯ No, I do not provide education to families
Who do you educate on the glycaemic impact of fat and protein?<br><br>Please select all that apply.	☐ Children that have fat and protein related postprandial glycaemic excursions<br>☐ Children that have unexplained postprandial glycaemic excursions<br>☐ Children/ families that ask<br>☐ Children meeting glycaemic targets<br>☐ Children not meeting glycaemic targets<br>☐ Children following a low carbohydrate diet<br>☐ Children consuming high fat and/ protein foods<br>☐ Children using CGM<br>☐ Other
Please describe the other group that you educate on the glycaemic impact of fat and protein?	__________________________________
When do you usually provide education on the glycaemic impact of fat and protein?<br><br>Please select the most common answer.	◯ At diagnosis<br>◯ At the first clinic visit<br>◯ Within the first year of diagnosis<br>◯ When the family ask, because they've heard that fat and protein can impact BGL's<br>◯ When the family notice unexplained postprandial glycaemic excursions<br>◯ When a member of the team identifies problematic postprandial glycaemic excursions<br>◯ When a member of the team identifies fat and protein related postprandial glycaemic excursions<br>◯ Other
Please describe the other timepoint when you provide education on the glycaemic impact of fat and protein.	__________________________________

In your clinic, do you have a policy or procedure for delivering fat and protein education (i.e. what information should be provided, to whom, how and when)?	◯ Yes<br>◯ No
Please provide a brief description of your clinics' policy or procedure for delivering fat and protein education.	__________________________________
Are there any barriers to providing education on the glycaemic impact of fat and protein?	◯ Yes<br>◯ No
Please select up to 3 main barriers.	☐ There is not enough time during clinic appointments<br>☐ There is no clear policy or procedure on what to teach, to whom, when and how<br>☐ I do not know how to do it<br>☐ There are no/ not enough resources to support education of the family<br>☐ There is no/ not enough training for me<br>☐ It unnecessarily burdens the family<br>☐ The family are not interested<br>☐ It is not a priority for me<br>☐ It is not a priority for the diabetes team<br>☐ Other
Please describe the other main barrier/s to providing education on the glycaemic impact of fat and protein.	__________________________________
How would you rate your confidence in recommending strategies to manage the glycaemic impact of fat and/ protein?	◯ Extremely confident<br>◯ Very confident<br>◯ Moderately confident<br>◯ Not very confident<br>◯ Not at all confident
Where there is evidence of an impact of fat and protein on glycaemia do you recommend strategies to manage this?	◯ Always<br>◯ Often<br>◯ Sometimes<br>◯ Rarely<br>◯ Never
What are your reasons for NOT recommending strategies, where there is evidence of an impact of fat and protein on glycaemia?<br><br>Please select up to 3 main reasons.	☐ I do not have time to explain what to do<br>☐ The child and/family are not interested<br>☐ The family is already overwhelmed<br>☐ I am not sure what the best strategy is to recommend<br>☐ I do not want to seem to be encouraging unhealthy foods<br>☐ I am worried the child may experience hypoglycaemia<br>☐ The child and/ family is worried about hypoglycaemia<br>☐ Other
Please describe the other reason for not recommending stratergies when there is evidence of an impact of fat and protein on glycaemia?	__________________________________

Which of the following strategies do you most often recommend to manage fat and protein in children using insulin pump therapy?<br><br>Please select up to 2 main strategies.	☐ Give the insulin for food earlier than usual<br>☐ Give the insulin for food later than usual<br>☐ Correct more frequently after the meal<br>☐ Give the insulin for food in a combination bolus<br>☐ Increase the insulin for food<br>☐ Decrease the insulin for food<br>☐ Increase the basal insulin<br>☐ Decrease the basal insulin<br>☐ Avoid the food<br>☐ Limit the amount of food eaten<br>☐ Other
Please specify the other strategy you use to manage fat and protein in children using insulin pump therapy.	__________________________________
As a starting point, what dose increase do you recommend to manage fat and protein in children using insulin pump therapy (as a % of the insulin dose for carbohydrate)?	0% 25% 50%<br>*(Place a mark on the scale above)*
What is the maximum dose increase that you recommend to manage fat and protein in children using insulin pump therapy (as a % of the insulin dose for carbohydrate)?	0% 30% 60%<br>*(Place a mark on the scale above)*
As a starting point, what combination bolus split (%:%) do you recommend to manage fat and protein in children using insulin pump therapy?	◯ 20:80<br>◯ 30:70<br>◯ 40:60<br>◯ 50:50<br>◯ 60:40<br>◯ 70:30<br>◯ 80:20
Which of the following strategies do you most often recommend to manage fat and protein in children using multiple daily injections?<br><br>Please select up to 2 main strategies.	☐ Give the insulin for food earlier than usual<br>☐ Give the insulin for food later than usual<br>☐ Correct more frequently after the meal<br>☐ Give the insulin for food in a split dose (2 injections)<br>☐ Increase the insulin for food<br>☐ Decrease the insulin for food<br>☐ Increase the long-acting insulin<br>☐ Decrease the long-acting insulin<br>☐ Avoid the food<br>☐ Limit the amount of food eaten<br>☐ Use regular insulin rather than rapid-acting or ultra-rapid acting insulin<br>☐ Use ultra-rapid acting insulin rather than rapid-acting insulin<br>☐ Other
Please specify the other strategy you use to manage fat and protein in children using multiple daily injections.	__________________________________

As a starting point, what dose increase do you recommend to manage fat and protein in children using multiple daily injections (as a % of the insulin dose for carbohydrate)?	0% 25% 50% *(Place a mark on the scale above)*
What is the maximum dose increase that you recommend to manage fat and protein in children using multiple daily injections (as a % of the insulin dose for carbohydrate)?	0% 30% 60% *(Place a mark on the scale above)*
How do you usually recommend the insulin dose is split (%:%) in children using multiple daily injections?	__________________________
Do you recommend strategies for managing fat and protein in children using hybrid closed loop therapy?	◯ Yes ◯ No
Which of the following strategies do you most often recommend to manage fat and protein in children using hybrid closed loop therapy? Please select up to 2 main strategies.	☐ Enter fake carbs on top of what the child is planning to eat ☐ Enter fake carbs later on ☐ Split the carb entry ☐ Eat less food ☐ Let auto-corrections take care of it ☐ Other
Please describe the other strategy you recommend to manage fat and protein in children using hybrid closed loop therapy.	__________________________
Do they strategies that you recommend to manage fat and protein differ depending on the specific type of hybrid closed loop pump?	◯ Yes ◯ No
Do you have any comments about how the strategies you recommend to manage fat and protein differ depending on the specific type of hybrid closed loop pump? Please leave here.	__________________________________
What are your reasons for NOT recommending strategies to manage fat and protein in children using hybrid closed loop therapy? Please select up to 2 main reasons.	☐ The algorithm takes care of fat and protein excursions ☐ I am not sure what strategies to recommend ☐ I am worried the child may experience hypoglycaemia ☐ The child and/ family is worried about hypoglycaemia ☐ The child and/ family are not interested ☐ It undermines the education we provide around trusting the algorithm ☐ Not many children in the clinic use auto-mode ☐ Other
Please specify the other reason/s why you do not recommend insulin strategies for managing fat and protein in children using hybrid closed loop therapy?	__________________________

What would enable you to provide fat and protein education and management strategies as part of routine practice?<br><br>Please select up to 3 main enablers.	☐ Training on what education to provide and how to deliver<br>☐ Resources to assist with delivering education<br>☐ Acknowledgement as a priority within the diabetes team<br>☐ More scientific evidence (research) around what strategies are most efficacious<br>☐ A clear procedure to guide fat and protein education<br>☐ Evidence that fat and protein education is important to families<br>☐ Other
Please specify the other enabler that would help you to provide fat and protein education as part of routine practice.	__________________________________

You are almost done! This is the final section of the survey. This section focuses on the use of CGM to identify the impact of foods, CGM targets and your interpretation of the CGM trace. It is estimated that this section will take less than 10 minutes to complete.

Do you use CGM to educate families on the glycaemic impact of different foods and/ food behaviours?

◯ Yes
◯ No

Using CGM, what foods and/ food behaviours do you educate families on?

Please select all that apply.

☐ High GI foods
☐ Fat and/ protein foods
☐ Grazing/ continuous snacking
☐ Supper
☐ Over-treating hypoglycaemia
☐ Missing an insulin dose for food
☐ Giving an insulin dose for food late
☐ Other

Please describe the other foods and/ food behaviours that you educate families on using CGM.

____

Are there any barriers to using CGM to educate families on the glycaemic impact of different foods and/ food behaviours?

◯ Yes
◯ No

Please select up to 3 main barriers.

☐ There is not enough time during clinic appointments
☐ I do not know how to interpret the CGM trace
☐ There is no clear policy or procedure on what to teach, to whom, when and how
☐ There are no resources to support education of the family
☐ It is too complex for most to understand
☐ It unnecessarily burdens the family
☐ The family are not interested
☐ The child has unstable glucose levels
☐ There is insufficient CGM data available
☐ It is not a priority for me
☐ It is not a priority for the diabetes team
☐ Other

Do you use CGM to guide adjustments to the insulin strategy for fat and protein?

◯ Yes
◯ No

Using the CGM trace, how do you know when to give additional insulin for a high fat and protein meal?

____

Using the CGM trace, how do you know when the insulin dose split needs adjusting AND additional insulin is needed for a high fat and protein meal?

____

How would you rate your confidence in identifying fat and protein related glycaemic excursions on the CGM trace?

◯ Extremely confident
◯ Very confident
◯ Moderately confident
◯ Not very confident
◯ Not at all confident

How would you rate your confidence in using the CGM trace to guide adjustments to the insulin dosing strategy for fat and protein?

◯ Extremely confident
◯ Very confident
◯ Moderately confident
◯ Not very confident
◯ Not at all confident

In your clinic, do children using CGM have a target for the peak postprandial glucose level?	◯ Yes, all children using CGM have a standard target for the peak postprandial glucose level<br>◯ Yes, we set individual targets for children using CGM for the peak postprandial glucose level<br>◯ No, we encourage children using CGM to strive for the lowest possible peak postprandial glucose level without hypoglycaemia<br>◯ No, we don't specifically discuss the peak postprandial glucose level with children using CGM
What is your clinics standard target for the peak postprandial glucose level?	__________________________________
What is the typical target range for the peak postprandial glucose level (min-max)?	__________________________________
Following a meal consisting predominately of carbohydrate when would you expect to see the peak glucose level on the CGM trace?	◯ 0-60 min<br>◯ 60-90 min<br>◯ 90-120 min<br>◯ 120-180 min<br>◯ I don't know
Following a meal high in fat and protein when would you expect to see the peak glucose level on the CGM trace?	◯ 0-60 min<br>◯ 60-120 min<br>◯ 120-180 min<br>◯ >180 min<br>◯ I don't know
Following a meal consisting predominately of carbohydrate what would you consider to be the target peak glucose excursion?	◯ < 3 mmol/L<br>◯ 3-5 mmol/L<br>◯ 5-8 mmol/L<br>◯ >8 mmol/L<br>◯ I don't know
Looking at the CGM trace below, what would you attribute the event circled in red to?	◯ High fat and protein meal<br>◯ High glycaemic index meal<br>◯ Over-treatment of hypoglycaemia<br>◯ I don't know
Looking at the CGM trace below, what would you attribute the event circled in red to?	◯ High fat and protein meal<br>◯ High glycaemic index meal<br>◯ Over-treatment of hypoglycaemia<br>◯ I don't know
Would you be interested in training and resources for families to support fat and protein education?	◯ Yes<br>◯ Maybe<br>◯ No
What would be your preference for mode of training delivery?	◯ Online, self-paced<br>◯ Online, live<br>◯ Combination of online self-paced and live (i.e. Q and A)<br>◯ In-person<br>◯ Combination of online self-paced and in-person<br>◯ No preference
